# Colonization with extended-spectrum and AmpC β-lactamase-producing Enterobacterales and bacteremia risk in patients with acute leukemia who receive induction chemotherapy without fluoroquinolone prophylaxis

**DOI:** 10.1128/aac.01738-25

**Published:** 2026-06-26

**Authors:** Kate Stoeckle, Ivo Sah Bandar, Liang Chen, Claire Douglass, Emily Davidson, Michael Hovan, Sierra Derti, Marisa La Spina, Gail J. Roboz, Justin Kaner, David Helfgott, Barry N. Kreiswirth, Lars F. Westblade, Michael J. Satlin

**Affiliations:** 1 Department of Medicine, Division of Infectious Diseases, Weill Cornell Medicine 639780 , New York, New York, USA; 2 Department of Pathology and Laboratory Medicine, Weill Cornell Medicine 207092 , New York, New York, USA; 3 School of Pharmacy and Pharmaceutical Sciences, University at Buffalo 12292 , Buffalo, New York, USA; 4 NewYork-Presbyterian/Weill Cornell Medical Center 159947 , New York, New York, USA; 5 Department of Medicine, Division of Internal Medicine, University of Maryland 195439, Baltimore, Maryland, USA; 6 Department of Medicine, Division of Internal Medicine, Allegheny Health Network 6596, Pittsburgh, Pennsylvania, USA; 7 Department of Medicine, Division of Hematology and Medical Oncology, Weill Cornell Medicine 537704 , New York, New York, USA; 8 Center for Discovery and Innovation, Hackensack Meridian Health 3139, Nutley, New Jersey, USA; 9 Department of Pediatrics, Division of Infectious Diseases, Weill Cornell Medicine 639780 , New York, New York, USA; University of Pennsylvania Perelman School of Medicine, Philadelphia, Pennsylvania, USA

**Keywords:** leukemia, bacteremia, ESBL, neutropenic

## Abstract

Neutropenic patients colonized with extended-spectrum β-lactamase-producing Enterobacterales (ESBL-E) frequently develop ESBL-E bacteremia while receiving fluoroquinolone prophylaxis. However, the impact of colonization with ESBL-E and AmpC β-lactamase-producing Enterobacterales (AmpC-E) in neutropenic patients who do not receive fluoroquinolone prophylaxis is unclear. To investigate this, we conducted a prospective study of patients with acute leukemia or myelodysplastic syndrome (MDS) receiving induction chemotherapy without antibacterial prophylaxis between November 2015 and January 2020. Stool or perianal swab specimens were collected from study participants within 5 days of initiating chemotherapy and underwent culture for ESBL-E and AmpC-E. Isolated organisms were identified and subjected to antimicrobial susceptibility testing and whole-genome sequencing. Bacteremia risk during neutropenia was compared between ESBL-E-colonized, AmpC-E-colonized, and non-colonized patients. We enrolled 159 patients, of whom 15 (9%) were colonized with ESBL-E and 17 (11%) with AmpC-E. Fourteen (93%) of 15 colonizing ESBL-E isolates were cefepime resistant, compared to 2 (12%) of 17 AmpC-E isolates. Three (20%) of 15 ESBL-E-colonized patients developed ESBL-E bacteremia (colonizing and bloodstream isolates were genetically identical for each patient), compared to 1 (1%) of 144 patients without ESBL-E colonization (*P* = 0.001). ESBL-E-colonized patients were also more likely to develop bacteremia due to ceftriaxone-susceptible Enterobacterales than non-colonized patients. AmpC-E-colonized patients did not have an increased risk of gram-negative bacteremia. In patients with acute leukemia or MDS who receive induction chemotherapy without fluoroquinolone prophylaxis, ESBL-E colonization is associated with increased risk of gram-negative bacteremia due to ESBL-E and ceftriaxone-susceptible organisms. Screening for ESBL-E colonization in this population could optimize empirical antibacterial therapy.

## INTRODUCTION

Patients with acute leukemia or myelodysplastic syndrome (MDS) who undergo induction chemotherapy are at high risk for gram-negative bacteremia because of mucositis and prolonged neutropenia (1, 2). When these patients develop fever or other signs of infection, immediate empirical antibacterial therapy with activity against enteric gram-negative bacteria and *Pseudomonas aeruginosa* is critical to prevent sepsis and death (3). However, the causative pathogen is typically unknown at the onset of symptoms, and if the infecting bacteria are resistant to the empirical regimen, delays in effective therapy can lead to life-threatening consequences.

Guidelines recommend initiating an anti-pseudomonal β-lactam such as cefepime or piperacillin-tazobactam as empirical therapy for fever in neutropenic patients (1, 4), and cefepime is most commonly used in the United States (5, 6). Unfortunately, these antimicrobial agents have limited effectiveness against many ceftriaxone-resistant Enterobacterales (CRO-R-E), particularly extended-spectrum β-lactamase-producing Enterobacterales (ESBL-E), which may be more effectively treated with a carbapenem (7–9). Neutropenic patients who develop ESBL-E bacteremia have higher mortality rates than those with bacteremia due to ceftriaxone-susceptible Enterobacterales, likely related to inadequate empirical therapies (10–12). However, universal empirical therapy with a carbapenem for all patients with fever and neutropenia is not an optimal strategy because it would promote the emergence of carbapenem-resistant Enterobacterales (CRE), pathogens that are associated with even higher mortality rates in neutropenic patients (13). Thus, strategies are needed to identify neutropenic patients at the highest risk for ESBL-E bacteremia, so that these patients can receive a carbapenem for empirical therapy, whereas lower-risk patients could receive cefepime or piperacillin-tazobactam.

We previously found that hematopoietic cell transplant (HCT) recipients who were colonized with ESBL-E prior to their transplant were at high risk of developing bacteremia from their colonizing strain during post-transplant neutropenia (14). Patients in this prior study received levofloxacin prophylaxis during neutropenia, and all breakthrough ESBL-E bacteremias were due to levofloxacin-resistant organisms. These findings suggest that screening for colonization with ESBL-E is a useful strategy to identify patients at high risk of ESBL-E bacteremia who would benefit from empirical therapy with a carbapenem in the setting of levofloxacin prophylaxis. However, it is unknown whether colonization with ESBL-E or other CRO-R-E, such as AmpC β-lactamase-producing Enterobacterales (AmpC-E), confers a high risk of bacteremia with resistant organisms when levofloxacin prophylaxis is not used and in other neutropenic patient populations. At our center, patients with acute myeloid leukemia (AML) or MDS who receive inpatient induction chemotherapy do not routinely receive fluoroquinolone prophylaxis. We therefore conducted this study to determine the prevalence and clinical significance of colonization with ESBL-E and AmpC-E in patients receiving induction chemotherapy for acute leukemia or MDS who do not receive fluoroquinolone prophylaxis.

## MATERIALS AND METHODS

### Study population and specimen collection

This prospective observational study was conducted at an 862-bed quaternary care academic medical center in New York City. We enrolled adult patients (age ≥18 years) with acute leukemia or MDS who were hospitalized for induction chemotherapy between November 2015 and January 2020 and who did not receive antibacterial prophylaxis. Exclusion criteria included receipt of fluoroquinolone prophylaxis, lack of a stool or perianal swab specimen within 5 days after initiation of induction chemotherapy, fewer than 7 days of neutropenia after induction chemotherapy, and discharge from the hospital within 7 days of collection of the first specimen. The study was approved by the Institutional Review Board of Weill Cornell Medicine (# 1504016114), and informed consent was obtained from study participants.

To assess for colonization with ESBL-E or AmpC-E, we collected stool or perianal swab specimens during the first 5 days after the initiation of induction chemotherapy and weekly thereafter until neutrophil recovery, defined as the first of two consecutive days with an absolute neutrophil count (ANC) ≥500 cells/µL. We used FisherBrand Commode Specimen Collection kits (Thermo Fisher Scientific, Waltham, Massachusetts) to collect stool specimens, and specimens were stored at 4°C immediately after collection. When stool specimens were not available, a perianal ESwab (Becton, Dickinson and Company [BD], Franklin Lakes, New Jersey) was collected and analyzed instead of the stool sample.

We collected data on patient demographics, including patient-reported race and ethnicity, type of underlying malignancy, chemotherapy, prior antimicrobial exposures, and history of multidrug-resistant bacterial infections. We also recorded all infections and antimicrobial therapies prior to recovery from neutropenia. Coagulase-negative staphylococci and other common skin commensals were only considered causes of bacteremia if isolated from ≥2 separate sets of blood cultures. Fever and neutropenia (FN) was defined as a temperature of >38.0°C with an ANC <500 cells/µL. At least two sets of blood cultures were obtained from febrile neutropenic patients prior to starting empirical antimicrobial therapy. CRO-R-E that were isolated from blood cultures were stored at −80°C for subsequent analysis.

### Microbiologic analysis

A 1 µL loopful of stool or 100 µL of Liquid Amies from perianal swabs was plated onto HardyCHROM ESBL chromogenic agar plates (Hardy Diagnostics, Santa Maria, California) within 24 hours of specimen collection. These agar plates were incubated at 37°C overnight in ambient air. Colonies identified after overnight incubation were subcultured onto BD trypticase soy agar II plates with 5% sheep blood and incubated overnight again in ambient air. The next day, colonies from these plates were identified using the MicroScan Walkaway *Plus* System with the Negative ID Type 2 Panel (Beckman Coulter, Brea, California) and underwent antimicrobial susceptibility testing using the Gram-Negative Panel 42 (Beckman Coulter). If the MicroScan system indicated an identification probability of <95%, organism identification was confirmed by matrix-assisted laser desorption/ionization time-of-flight mass spectrometry (Bruker, Billerica, Massachusetts). The interpretive criteria of the Clinical and Laboratory Standards Institute in 2025 were applied (15). Ceftriaxone-resistant *Escherichia coli*, *Klebsiella pneumoniae*, *Klebsiella oxytoca*, and *Proteus mirabilis* isolates underwent phenotypic testing for ESBL production using cefotaxime and ceftazidime disks, with and without clavulanate (15). The results of this phenotypic test were used to define ESBL production in these four species. Isolates belonging to other species of Enterobacterales or where the phenotypic ESBL test result was negative were considered AmpC-E based on the results of whole-genome sequencing (WGS). Results of screening for colonization with ESBL-E and AmpC-E were not reported to patient care teams, and thus these screening results did not impact choices of antimicrobial therapy.

### Sequencing of CRO-R-E isolates

To determine the antimicrobial resistance mechanisms of colonizing strains and the genetic relatedness of isolates, initial colonizing and bloodstream CRO-R-E isolates underwent WGS using the Illumina HiSeq platform (Illumina, San Diego, CA) with 2 × 150 base pair paired-end reads. Methods to sequence and assemble raw reads, as well as multilocus sequence typing, identification of resistance genes, and determination of single-nucleotide polymorphism (SNP) phylogenetic analysis, were performed as previously described (16).

### Statistical analysis

We determined the proportion of patients who were colonized with ESBL-E, AmpC-E, and not colonized with CRO-R-E at the initiation of induction chemotherapy. Factors associated with baseline ESBL-E and AmpC-E colonization were assessed by comparing characteristics of patients colonized with these organisms to those not colonized with CRO-R-E, using chi-square or Fisher’s exact test for categorical variables and the Wilcoxon rank-sum test for continuous variables. We then compared the cumulative incidence of developing ESBL-E and other bacteremia types prior to neutrophil recovery, as well as clinical outcomes between ESBL-E-colonized patients and those not colonized with CRO-R-E, and between AmpC-E-colonized patients and those not colonized with CRO-R-E. Among patients not initially colonized with ESBL-E or AmpC-E at baseline and who had a follow-up stool or perianal swab specimen collected, we assessed the frequency of ESBL-E or AmpC-E acquisition. Stata version 15.0 (StataCorp, College Station, Texas) was used for statistical analyses, and two-tailed *P* values ≤0.05 were considered statistically significant.

## RESULTS

### Patients and colonization status

Of 233 patients with acute leukemia or MDS enrolled, 159 were eligible for analysis. Patients were excluded for the following reasons: stool or swab specimen not collected within 5 days of starting chemotherapy (*n* = 28), receipt of levofloxacin prophylaxis during neutropenia (*n* = 28), <7 days of neutropenia (*n* = 17), and discharged within 7 days of first specimen collection (*n* = 1). The median patient age was 63 years (interquartile range [IQR] 49–72). Seventy-one (45%) patients were women, 47 (30%) were non-White, and 16 (10%) were Hispanic/Latino. One hundred and forty-one (89%) patients had AML or MDS, and 135 (85%) had a new diagnosis of leukemia. The most common chemotherapy regimen was an anthracycline plus cytarabine (63%), followed by decitabine alone (9%) and cytarabine plus venetoclax (6%). The median duration of neutropenia was 21 days (IQR: 17–29).

One hundred and two (64%) patients had a stool specimen analyzed as their baseline sample, and 57 (36%) had a perianal swab. Thirty-one (19%) patients were colonized with CRO-R-E at baseline, including 15 (9%) with an ESBL-E and 17 (11%) with an AmpC-E (Table 1). One patient was colonized with an ESBL-E, AmpC-E, and a carbapenemase-producing organism, and this was the only patient colonized with a CRE. Patients colonized with ESBL-E were older than those not colonized with CRO-R-E, were more likely to receive induction chemotherapy with cytarabine only, and were less likely to receive an anthracycline plus cytarabine. Patients colonized with AmpC-E were more likely to have acute lymphoblastic leukemia and to receive induction chemotherapy with hyperfractionated cyclophosphamide, vincristine, adriamycin, and dexamethasone than patients not colonized with CRO-R-E. Only one of 31 patients colonized with CRO-R-E had a prior positive culture for CRO-R-E.

**TABLE 1:** Baseline characteristics of patients with acute leukemia colonized with ESBL-E, AmpC-E, and not colonized with CRO-R-E^*c*^

Variables	Colonized with CRO-R-E		Not colonized with CRO-R-E (*n* = 128)	*P* value^*a*^ (ESBL-E)	*P* value^*b*^ (AmpC-E)
	ESBL-E-colonized (*n* = 15)	AmpC-E-colonized (*n* = 17)			
Demographics					
Age, years	70 (60–77)	61 (39–67)	63 (46–71)	**0.005**	0.43
Female gender	4 (27)	8 (47)	59 (46)	0.15	0.94
Race					
White	10 (67)	14 (82)	89 (70)	0.82	0.40
Black	2 (13)	2 (12)	22 (17)	1.00	0.74
Asian	2 (13)	1 (6)	9 (7)	0.32	1.00
Native Hawaiian or Pacific Islander	0	0	1 (1)	1.00	1.00
Not reported	1 (7)	0	7 (5)	0.60	1.00
Hispanic ethnicity					
Hispanic	3 (20)	3 (18)	11 (9)	0.17	0.21
Not Hispanic	12 (80)	14 (82)	116 (91)	0.20	0.39
Not reported	0	0	1 (1)	1.00	1.00
Acute leukemia type					
AML or MDS	13 (87)	13 (76)	116 (91)	0.64	0.10
ALL	1 (7)	3 (18)	3 (2)	0.36	**0.02**
APL	1 (7)	0	6 (5)	0.55	1.00
Mixed lineage	0	1 (6)	3 (2)	1.00	0.40
Disease status					
New diagnosis	12 (80)	14 (82)	109 (85)	0.70	0.72
Relapsed or refractory disease	3 (20)	3 (18)	19 (15)	0.70	0.72
Induction chemotherapy					
Anthracycline plus cytarabine	6 (40)	8 (47)	86 (67)	**0.04**	0.10
Decitabine alone	2 (13)	2 (12)	12 (9)	0.64	0.67
Venetoclax plus cytarabine	2 (13)	0	7 (5)	0.24	1.00
Hypomethylating agent plus venetoclax	1 (7)	1 (6)	5 (4)	0.49	0.53
ATRA plus arsenic trioxide	1 (7)	0	6 (5)	0.55	1.00
HyperCVAD	1 (7)	3 (18)	0	0.11	**0.001**
Cytarabine alone	2 (13)	1 (6)	1 (1)	**0.03**	0.22
Other regimen	0	2 (12)	11 (9)	0.61	0.65
Healthcare exposures within the previous 90 days					
Hospitalization	8 (53)	8 (47)	43 (34)	0.13	0.28
Receipt of any antibacterial agent	7 (47)	9 (53)	66 (52)	0.72	0.92
β-lactam agent	1 (7)	4 (24)	26 (20)	0.30	0.75
Fluoroquinolone	5 (33)	4 (24)	35 (27)	0.76	1.00
History of positive microbiologic tests for specified bacteria at any time prior to the study					
CRO-R-E	1 (7)	1 (6)	2 (2)	0.29	0.31
Fluoroquinolone-resistant Enterobacterales	1 (7)	2 (12)	2 (2)	0.29	0.07
Vancomycin-resistant enterococci	2 (13)	1 (6)	3 (2)	0.09	0.40
*Clostridioides difficile*	0	0	2 (2)	1.00	1.00

^*a*^ Comparison of ESBL-E-colonized patients to patients not colonized with CRO-R-E.

^*b*^ Comparison of AmpC-E-colonized patients to patients not colonized with CRO-R-E.

^*c*^ Data are presented as No. (% of total of column) or median (interquartile range). *P *values <0.05 are bolded. One patient was colonized with ESBL-E and AmpC-E. ALL, acute lymphoblastic leukemia; AML, acute myeloid leukemia; AmpC-E, AmpC β-lactamase-producing Enterobacterales; APL, acute promyelocytic leukemia; ATRA, all-trans retinoic acid; CRO-R-E, ceftriaxone-resistant Enterobacterales; ESBL-E, extended-spectrum β-lactamase-producing Enterobacterales; HyperCVAD, hyperfractionated cyclophosphamide, vincristine, doxorubicin, and dexamethasone; IQR, interquartile range; MDS, myelodysplastic syndrome.

### Colonizing strains of ESBL-E and AmpC-E

**Fig 1:**
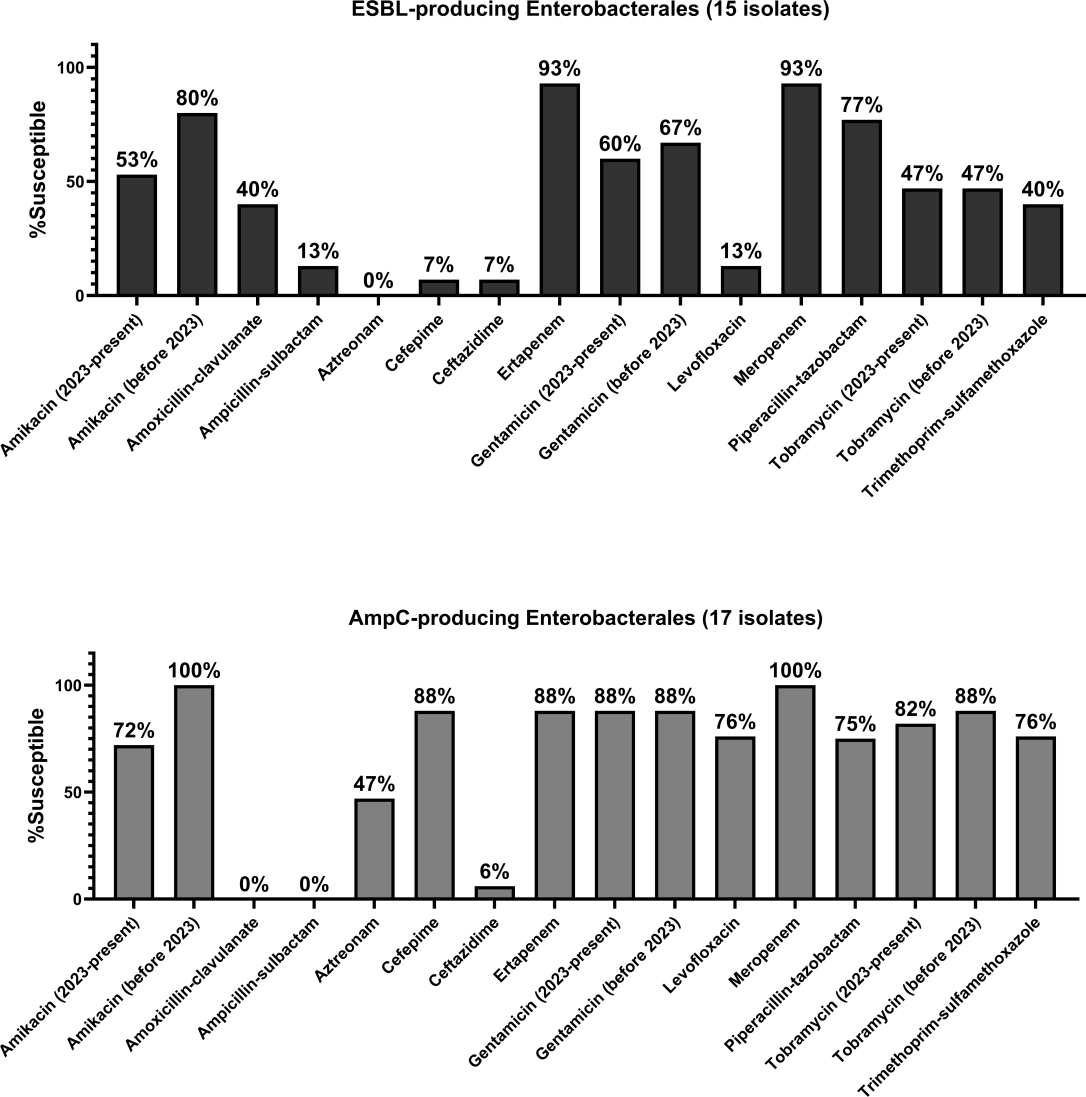
Antimicrobial susceptibilities of colonizing ESBL-E and AmpC-E isolates. Antimicrobial susceptibility testing was performed using MicroScan NM43 panels and interpreted using Clinical and Laboratory Standards Institute M100 S35 breakpoints (15). All isolates were resistant to ampicillin, cefazolin, and ceftriaxone. Cefepime % susceptible includes isolates that were susceptible and susceptible dose-dependent.

Of 15 ESBL-E-colonizing strains, 13 were *E. coli,* and two were *K. pneumoniae* (Table 2). All 15 strains harbored *bla*_CTX-M_, of which 9 were *bla*_CTX-M-15_. Six of the 13 ESBL-producing *E. coli* strains were multilocus sequence type 131. Of the 17 isolates considered to be AmpC-E, six were *E. coli*, three were *Citrobacter freundii*, two each were *Hafnia alvei*, *Klebsiella aerogenes*, and *K. pneumoniae*, and one each was *Citrobacter youngae* and *Morganella morganii* (Table 2). Fifteen of these 17 AmpC-E isolates underwent sequencing, and the AmpC β-lactamase genes harbored by these isolates were *bla*_CMY_ (*n* = 11), *bla*_DHA_ (*n* = 2), and *bla*_ACC_ (*n* = 2). The proportions of ESBL-E and AmpC-E isolates susceptible to ceftazidime, piperacillin-tazobactam, and meropenem were similar (Fig. 1). AmpC-E isolates were more likely to be susceptible to cefepime (88% vs. 7%) and levofloxacin (76% vs. 13%) than ESBL-E isolates.

**TABLE 2:** Characteristics of colonizing CRO-R-E isolates^*a*^

Characteristic	No. of isolates
ESBL-producing Enterobacterales (*n* = 15)	
Species and sequence types	
*Escherichia coli*	13
ST131	6
ST1193	2
Other sequence type^*b*^	5
*Klebsiella pneumoniae^c^*	2
β-lactamase genes	
ESBL genes	15
*bla*_CTX-M-15_	9^*d*^
*bla*_CTX-M-55_	3
Other *bla*_CTX-M_	3^*e*^
Other β-lactamase genes	12
*bla*_TEM-1_	9
*bla*_OXA-1_	6
*bla*_SHV-11_	1
AmpC-producing Enterobacterales (*n*=17)	
Species	
*Escherichia coli*	6^*f*^
*Citrobacter freundii*	3
*Hafnia alvei*	2
*Klebsiella aerogenes*	2
*Klebsiella pneumoniae*	2
*Citrobacter youngae*	1
*Morganella morganii*	1
β-lactamase genes (sequencing performed on 15 of 17 isolates)	
AmpC-encoding genes	15
*bla*_CMY_	11
*bla*_DHA_	2
*bla*_ACC_	2
Other β-lactamase genes	5
*bla*_TEM-1_	4
*bla*_TEM-176_	1
Carbapenemase-producing Enterobacterales (*n*=1)	
*Klebsiella pneumoniae* (*bla*_KPC-3_)	1^*g*^

^*a*^ CRO-R-E, ceftriaxone-resistant Enterobacterales; ESBL, extended-spectrum β-lactamase.

^*b*^ Other ESBL-producing *E. coli *sequence types were ST405 (*n *= 1), ST648 (*n *= 1), ST2171 (*n *= 1), ST6756 (*n *= 1), and ST unknown (*n *= 1).

^*c*^ ESBL-producing *K. pneumoniae *sequence types were ST15 (*n *= 1) and ST17 (*n *= 1).

^*d*^ One isolate harbored *bla*_CTX-M-55 _and the ESBL gene *bla*_SHV-28_.

^*e*^ Other CTX-M producing genes were *bla*_CTX-M-14_ (*n *= 1), *bla*_CTX-M-27_ (*n *= 1), and unknown *bla*_CTX-M _type (*n *= 1).

^*f*^ AmpC-producing *E. coli *sequence types were ST68 (*n *= 1), ST354 (*n *= 1), ST648 (*n *= 1), ST847 (*n *= 1), ST977 (*n *= 1), and ST1196 (*n *= 1).

^*g*^ This organism was ST258 and also harbored *bla*_SHV-11 _and *bla*_OXA-9_.

### Risk of bacteremias and other outcomes based on colonization status

**Fig 2:**
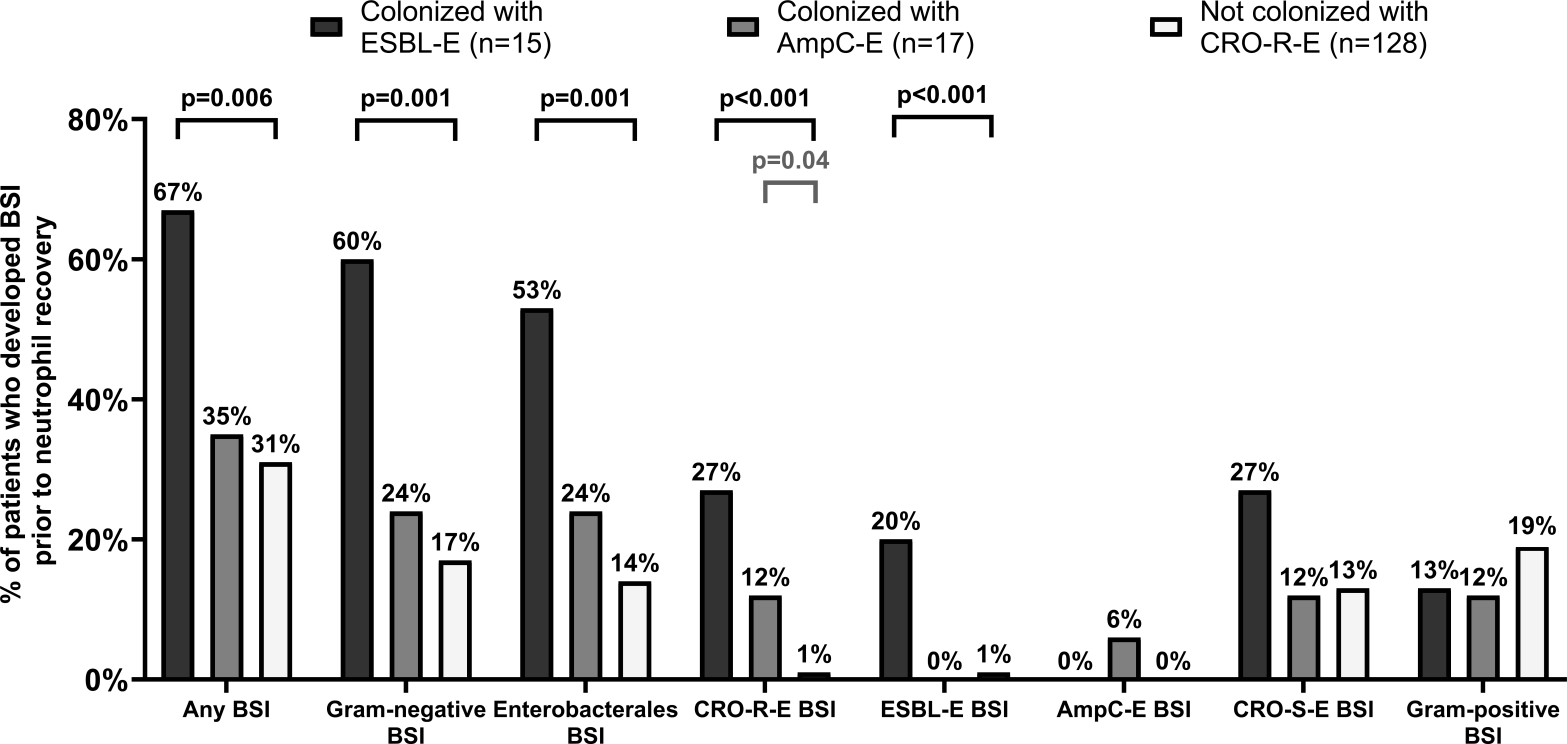
Bacteremia risk prior to neutrophil recovery in patients colonized with ESBL-E, colonized with AmpC-E, and not colonized with CRO-R-E at baseline.

**Fig 3:**
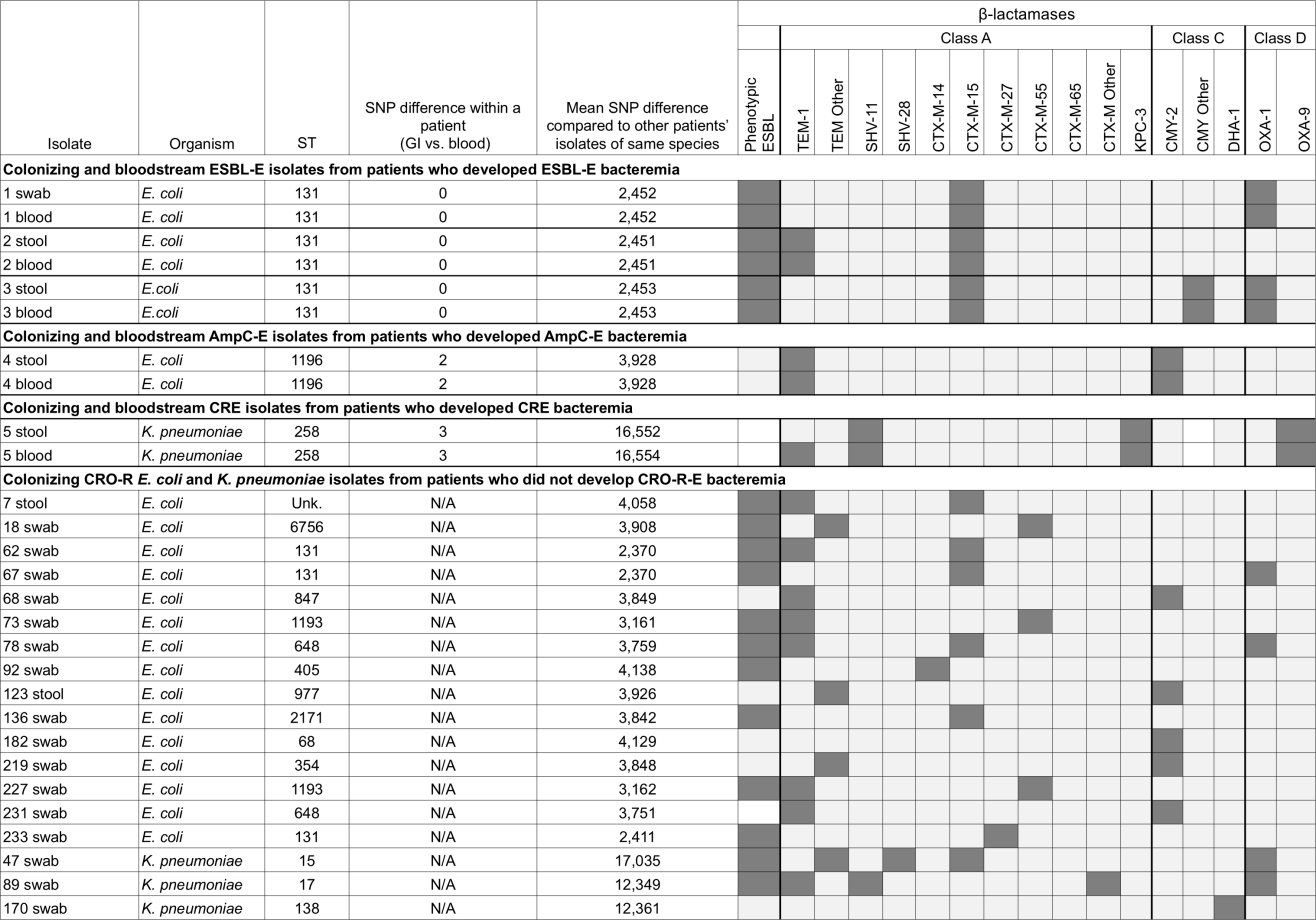
Comparison of organisms, multilocus sequence types, SNP differences, phenotypic ESBL testing based on disk diffusion with and without clavulanate (15), and β-lactamase genes among colonizing (stool or swab) and bloodstream CRO-R-E isolates. Results are paired for colonized patients who developed CRO-R-E bloodstream infections. GI, gastrointestinal; N/A, not applicable; SNP, single-nucleotide polymorphism; ST, sequence type.

Three (20%) of the 15 ESBL-E-colonized patients developed ESBL-E bacteremia prior to neutrophil recovery, compared with one of the 144 patients without baseline ESBL-E colonization (*P =* 0.001, Fig. 2). Two of these three patients received empirical therapy with piperacillin-tazobactam, and one received meropenem. In all three ESBL-E-colonized patients who developed ESBL-E bacteremia, the colonizing strain was a ST 131 *E. coli* that harbored *bla*_CTX-M-15_, and there were no SNP differences between the colonizing and bloodstream isolates (Fig. 3). In contrast, there were thousands of SNP differences between these *E. coli* isolates and CRO-R-*E. coli* isolates recovered from other patients. ESBL-E-colonized patients were not only more likely to develop ESBL-E bacteremia compared to other patients, but they were also more likely to develop Gram-negative bacteremia in general. Four (27%) of 15 ESBL-E colonized patients developed gram-negative bacteremia due to ceftriaxone-susceptible organisms (Fig. 2).

One (6%) of the 17 AmpC-E-colonized patients developed AmpC-E bacteremia prior to neutrophil recovery, compared to none of the 142 patients without baseline AmpC-E colonization (*P*=0.11; Fig. 2). In this case, the colonizing and bloodstream isolate was a ST 1196 *E. coli* that harbored *bla*_CMY-2_ and *bla*_TEM-1_, and there were only two SNP differences between the colonizing and bloodstream isolate (Fig. 3). This patient was empirically treated with piperacillin-tazobactam. Additionally, one patient who was colonized with an ESBL-producing *E. coli*, AmpC-producing *Citrobacter freundii*, and *Klebsiella pneumoniae* carbapenemase (KPC)-producing *K. pneumoniae* developed bacteremia due to their KPC-producing organism. Unlike ESBL-E-colonized patients, AmpC-E-colonized patients did not have an increased risk of gram-negative bacteremia compared to non-colonized patients.

Fever and neutropenia occurred in 14 (93%) of 15 ESBL-E-colonized patients, 13 (76%) of 17 AmpC-E-colonized patients, and 102 (80%) of 128 patients not colonized with CRO-R-E (Table 3). There were no significant differences in the number of days from the start of induction chemotherapy until FN among these three groups. Piperacillin-tazobactam was the most common empirical agent administered for FN, and it was used in 89 (70%) of 128 patients with FN. Seven (50%) of 14 ESBL-E colonized patients had gram-negative bacteremia during their first episode of FN, compared to only 3 (23%) of 13 AmpC-E-colonized patients and 13 (13%) of 102 non-colonized patients. Of the seven ESBL-E-colonized patients who developed gram-negative bacteremia during initial FN, four were due to ceftriaxone-susceptible Enterobacterales, two due to ceftriaxone-resistant ESBL-E (one with a *Pseudomonas aeruginosa* co-infection) and one due to a ceftriaxone-intermediate *Acinetobacter nosocomialis*. In contrast, the risk of gram-negative bacteremia during the first episode of FN was not increased in AmpC-E-colonized patients compared to patients without CRO-R-E colonization. Among the 102 patients not colonized with CRO-R-E who developed FN, only one had bacteremia due to a CRO-R-E (an ESBL-E) during the initial FN episode, and this occurred 34 days after the initiation of chemotherapy.

**TABLE 3:** Comparison of the incidence of fever and neutropenia, etiologies of bacteremia during the first febrile neutropenic episode, and outcomes in patients colonized with ESBL-E, AmpC-E, and not colonized with CRO-R-E upon initiation of chemotherapy^*a*^

Variable	ESBL-E-colonized (*n* = 15)	AmpC-E-colonized (*n* = 17)	Not colonized with CRO-R-E (*n* = 128)	*P* value^*b*^ (ESBL-E)	*P* value^*c*^ (AmpC-E)
Fever and neutropenia (FN)	14 (93)	13 (76)	102 (80)	0.30	0.75
Days from the start of induction chemotherapy until FN	8 (6-12)	10 (7-12)	8 (6-12)	0.90	0.14
Bacteremia at the time of FN^*d*^	8/14 (57)	3/13 (23)	20/102 (20)	**0.005**	0.72
Gram-positive bacteremia	1/14 (7)	0/13 (0)	6/102 (6)	1.00	1.00
Gram-negative bacteremia	7/14 (50)	3/13 (23)	13/102 (13)	**0.003**	0.39
ESBL-E^*e*^	2/14 (14)	0/13 (0)	1/102 (1)	**0.038**	1.00
AmpC-E	0/14 (0)	1/13 (8)^*f*^	0/102 (0)	N/A	0.11
Ceftriaxone-susceptible Enterobacterales	4/14 (29)	2/13 (15)	10/102 (10)	0.066	0.62
Microbiologically documented bacterial infection other than bacteremia^*g*^	2 (13)	1 (6)	12 (9)	0.62	1.00
ICU admission prior to neutrophil recovery	0	1 (6)	12 (9)	0.37	1.00
Septic shock prior to neutrophil recovery^*h*^	0	1 (6)	6 (5)	1.00	1.00
Days of hospitalization from the start of chemotherapy, median (IQR)	29 (24-43)	28 (24-45)	28.5 (24-35)	0.52	0.56
30-day mortality	0	1 (6)	9 (7)	0.60	1.00
100-day mortality	4 (27)	3 (18)	16 (13)	0.23	0.47

^*a*^ Data are presented as No. (% of total) or median (IQR). *P *values <0.05 are bolded. AmpC-E, AmpC β-lactamase-producing Enterobacterales; ESBL-E, extended-spectrum β-lactamase-producing Enterobacterales; ICU, intensive care unit; IQR, interquartile range; N/A, not applicable.

^*b*^ Comparison of ESBL-E-colonized patients to patients not colonized with CRO-R-E.

^*c*^ Comparison of AmpC-E-colonized patients to patients not colonized with CRO-R-E.

^*d*^ The denominators for bacteremia analyses are the number of patients with FN.

^*e*^ All were due to CTX-M-producing *E. *coli. One of the two ESBL-colonized patients who had ESBL-E bacteremia was empirically treated with piperacillin-tazobactam, and the other was empirically treated with meropenem. The patient not initially colonized with CRO-R-E who developed ESBL-E bacteremia was empirically treated with piperacillin-tazobactam.

^*f*^ The AmpC-E-colonized patient who developed AmpC-E bacteremia due to CMY-2-producing *E. coli* was empirically treated with piperacillin-tazobactam.

^*g*^ Microbiologically documented bacterial infections other than bacteremia were *Clostridioides difficile *infection (*n *= 10), enterococcal urinary tract infection (*n *= 2), a polymicrobial perirectal abscess (*n *= 1), a skin infection due to *Stenotrophomonas maltophilia *(*n *= 1), and an intestinal infection due to Shiga toxin-producing *Escherichia coli *(*n *= 1).

^*h*^ Septic shock was defined as hypotension requiring vasopressor support in the setting of a microbiologically documented infection without a clear alternate explanation for shock.

There were no significant differences in microbiologically documented infections other than bacteremia, intensive care unit admissions, incidence of septic shock, duration of hospitalization, or 30- or 100-day mortality between ESBL-E-colonized, AmpC-E-colonized, and non-colonized patients. Of the 128 patients who were not initially colonized with CRO-R-E, 111 had subsequent samples (a median of 2 additional samples) to determine whether they acquired CRO-R-E prior to recovery from neutropenia. Sixteen (14%) of these 111 patients acquired CRO-R-E (19 isolates in total), and none of these 16 patients developed CRO-R-E bacteremia. The acquired CRO-R-E isolates were *K. pneumoniae* (*n* = 5), *Enterobacter cloacae* (*n* = 4), *Hafnia alvei* (*n* = 4), *Citrobacter freundii* (*n* = 2), and *E. coli*, *Klebsiella aerogenes*, *Klebsiella oxytoca*, and *Morganella morganii* (*n* = 1, each). Only 3 (16%) of these 19 acquired isolates were ESBL producers.

## DISCUSSION

This study prospectively determined the prevalence and clinical impact of colonization with ESBL-E and AmpC-E in hospitalized patients with acute leukemia and MDS who were receiving induction chemotherapy without fluoroquinolone prophylaxis. We found that 9% of patients were colonized with ESBL-E and 11% were colonized with AmpC-E upon initiation of induction chemotherapy. Patients colonized with ESBL-E were more likely to develop gram-negative bacteremia during neutropenia than patients not colonized with CRO-R-E. These infections were due to both CRO-susceptible Enterobacterales and cefepime-resistant ESBL-E that were genetically identical to the colonizing strain. In contrast, patients colonized with AmpC-E did not have an increased risk of gram-negative bacteremia compared to patients not colonized with CRO-R-E, and only one AmpC-E-colonized patient developed bacteremia due to an AmpC-E. Of the 128 patients not colonized with ESBL-E or AmpC-E, only one developed CRO-R-E bacteremia.

Our finding that approximately 10% of patients with acute leukemia or MDS were colonized with ESBL-E aligns with the previously identified colonization prevalence in patients undergoing HCT at the same center and is similar to the 10%–20% colonization prevalence in studies of patients with hematologic malignancies from European centers (16–19). The ESBL-E colonization prevalence in this study is notable because most patients were only recently diagnosed with their malignancy, and only approximately one-half had a recent hospitalization or exposure to an antimicrobial agent. These findings highlight that ESBL-E, particularly *E. coli* of sequence type (ST) 131, circulates in the community in the United States and elsewhere (20, 21). In contrast to our study of HCT recipients, we found that Amp-C-E colonization was just as common as ESBL-E colonization, particularly due to *E. coli* that harbored the plasmid-encoded AmpC β-lactamase CMY. Moreover, we identified organisms that harbored DHA and ACC AmpC β-lactamases, highlighting the variety of CRO resistance mechanisms among Enterobacterales isolates harbored by neutropenic patients.

Few clinical risk factors for ESBL-E or AmpC-E colonization were identified in our study. Thus, depending on a risk factor-based approach to identify leukemia patients who harbor these organisms is unlikely to reliably identify colonized patients. Moreover, only one patient had a history of a prior clinical culture positive for CRO-R-E. Thus, relying on a review of prior microbiology results is also unlikely to be a sensitive method of detecting colonization. Instead, screening for gut colonization is necessary to identify patients with leukemia who are colonized with these organisms.

We found different clinical implications of being colonized with ESBL-E and AmpC-E in regard to bacteremia risk during neutropenia in this cohort of leukemia patients who did not receive fluoroquinolone prophylaxis. ESBL-E-colonized patients were more likely to develop gram-negative bacteremia than patients not colonized with CRO-R-E (60% vs. 17%), and some cases were due to their colonizing ESBL-E strains. In contrast, AmpC-E-colonized patients did not have a significantly increased risk of gram-negative bacteremia (24% vs. 17%), and only one colonized patient developed bacteremia due to their colonizing AmpC-E strain. Moreover, only 7% of colonizing ESBL-E isolates were susceptible to cefepime, compared to 88% of AmpC-E isolates. This is relevant because cefepime is the most common antimicrobial agent used for empirical treatment of FN in the United States (6). Thus, ESBL-E colonized patients who develop ESBL-E bacteremia are unlikely to receive active empirical therapy when using cefepime, whereas cefepime is appropriate for AmpC-E-colonized patients who develop AmpC-E bacteremia. In our center, where piperacillin-tazobactam is the primary empirical agent for FN, two of the three ESBL-colonized patients who developed ESBL-E bacteremia received suboptimal empirical therapy with piperacillin-tazobactam (9), although none developed septic shock.

The risk of ESBL-E bacteremia during neutropenia in ESBL-E-colonized patients was 20% in this cohort. This is consistent with prior studies from Europe and Latin America, which have reported a 2%–22% ESBL-E bacteremia risk in ESBL-E-colonized neutropenic patients when fluoroquinolone prophylaxis is not used (17, 19, 22). Although this bacteremia risk is lower than the 31% risk of ESBL-E bacteremia in colonized patients undergoing HCT who received fluoroquinolone prophylaxis at our center (14), these data suggest that ESBL-E-colonized neutropenic patients are still at risk of ESBL-E bacteremia even in the absence of fluoroquinolone prophylaxis. Moreover, these ESBL-E bacteremias typically occurred during the first episode of FN and were caused by the colonizing strain. Thus, screening for ESBL-E colonization and administering a carbapenem for fever and neutropenia may be a reasonable strategy even in the absence of the selective pressure of fluoroquinolone prophylaxis. However, in the absence of fluoroquinolone prophylaxis, ESBL-E-colonized leukemia patients were just as likely, if not more likely, to develop bacteremia due to a CRO-susceptible Enterobacterales. For example, during the first episode of FN in this cohort, four out of six gram-negative bloodstream isolates in ESBL-E colonized patients were CRO-susceptible, compared to two out of 11 in our HCT cohort who received fluoroquinolone prophylaxis (14). Thus, identifying ESBL-E colonization in neutropenic patients who do not receive fluoroquinolone prophylaxis may have less predictive value as to the etiologies of gram-negative bacteremia during FN than in patients who receive fluoroquinolone prophylaxis.

Another important finding from this study is that only one of 128 patients not colonized with CRO-R-E at baseline developed an ESBL-E or AmpC-E bacteremia, and this single bacteremia event occurred after >30 days of neutropenia. Although 14% of patients who were not colonized with CRO-R-E at baseline acquired a CRO-R-E during their episode of neutropenia, none of these acquisition events led to CRO-R-E bacteremia. Thus, patients who are not colonized with CRO-R-E upon initiation of induction chemotherapy for acute leukemia or MDS are unlikely to need carbapenem therapy for FN, and these patients can safely be administered cefepime or piperacillin-tazobactam. This illustrates how screening for colonization with ESBL-E or AmpC-E can guide risk stratification and antimicrobial stewardship in neutropenic patients.

This study has limitations. First, it was conducted at a single center, which may limit generalizability to other centers. Second, the study included only inpatients who were receiving induction chemotherapy. Additional studies are needed to determine whether the risk of bacteremia would differ in patients who received outpatient induction chemotherapy regimens. Third, we did not perform a broth enrichment step when culturing ESBL-E, and this has been shown to increase the yield of detecting ESBL-E in culture (23). Thus, it is possible that we underestimated the ESBL-E and AmpC-E colonization prevalence. However, our direct plating method had sufficient sensitivity to detect patients at risk of ESBL-E bacteremia. Fourth, some patients in our cohort had a perianal swab specimen instead of a stool specimen at baseline. Nonetheless, we have previously demonstrated that perianal swabs have similar sensitivity to stool specimens for the detection of ESBL-E (24), and thus, we do not think the use of perianal swabs significantly impacted the colonization prevalence findings. Lastly, we only identified 15 ESBL-E colonized patients, and thus our estimate of the risk of ESBL-E bacteremia in colonized patients has limited precision. We encourage additional studies of larger numbers of ESBL-E-colonized leukemia patients to better define this risk.

In conclusion, this study supports the potential role of screening for colonization with ESBL-E to guide empirical therapy in patients with acute leukemia or MDS who do not receive fluoroquinolone prophylaxis during induction chemotherapy. Additional studies are needed to further define bacteremia risk in ESBL-E-colonized patients who do not receive fluoroquinolone prophylaxis, and to more clearly delineate the benefits and limitations of screening for ESBL-E colonization in this patient population.
